# Biological Aging Acceleration Due to Environmental Exposures: An Exciting New Direction in Toxicogenomics Research

**DOI:** 10.3390/genes15010016

**Published:** 2023-12-21

**Authors:** Sudipta Dutta, Jaclyn M. Goodrich, Dana C. Dolinoy, Douglas M. Ruden

**Affiliations:** 1 Department of Veterinary Integrative Biosciences, College of Veterinary Medicine and Biomedical Sciences, Texas A&M University, College Station, TX 77843, USA; sdutta@cvm.tamu.edu; 2 Department of Environmental Health Sciences, School of Public Health, University of Michigan, Ann Arbor, MI 48109, USA; gaydojac@umich.edu (J.M.G.); ddolinoy@umich.edu (D.C.D.); 3 Department of Nutritional Sciences, School of Public Health, University of Michigan, Ann Arbor, MI 48109, USA; 4 C. S. Mott Center for Human Health and Development, Department of Obstetrics and Gynecology, Institute of Environmental Health Sciences, Wayne State University, Detroit, MI 48202, USA

**Keywords:** biological clocks, epigenetic clocks, toxicology, toxicogenomics, DNA methylation

## Abstract

Biological clock technologies are designed to assess the acceleration of biological age (B-age) in diverse cell types, offering a distinctive opportunity in toxicogenomic research to explore the impact of environmental stressors, social challenges, and unhealthy lifestyles on health impairment. These clocks also play a role in identifying factors that can hinder aging and promote a healthy lifestyle. Over the past decade, researchers in epigenetics have developed testing methods that predict the chronological and biological age of organisms. These methods rely on assessing DNA methylation (DNAm) levels at specific CpG sites, RNA levels, and various biomolecules across multiple cell types, tissues, and entire organisms. Commonly known as ‘biological clocks’ (B-clocks), these estimators hold promise for gaining deeper insights into the pathways contributing to the development of age-related disorders. They also provide a foundation for devising biomedical or social interventions to prevent, reverse, or mitigate these disorders. This review article provides a concise overview of various epigenetic clocks and explores their susceptibility to environmental stressors.

## 1. Introduction

Everyone ages, but biologically not at the same pace. Aging is quantified based on two parameters—chronological age (C-age) is the amount of time that has elapsed from birth to a given date, and biological age (B-age) is the measure of the apparent age of an organ or tissue based on several factors discussed in this review. Individuals with the same C-age can have different health status, and therefore C-age offers limited information regarding the biological pathways underlying aging [1]. That led to the development of the concept of “biological age” which can accurately reflect phenotypic characteristics such as “functional frailty” and molecular level markers such as DNAm [2,3,4,5]. Over the past decade, aging has become a critical public health concern because of several socio-economic implications [6]. The unprecedented increase in world’s aging population will continue to rise globally in the next few decades and by 2050, the number of people aged 60 and older would reach 21% [7]. This varies from 9.3% in Africa (low) to 36.5% in Japan (high) and 21.4% in the USA in 2050 [8]. After adolescent growth and development, there is a progressive decline in tissue and organ function along with an exponential rise in mortality rates, doubling roughly every 7–8 years after puberty [9]. The prevalence of significant chronic conditions, such as cardiovascular disease, cancer, diabetes, and Alzheimer’s disease, increases exponentially as individuals age [6]. This is primarily caused by a progressive gradual accumulation of deleterious changes to normal metabolism at the molecular, cellular, and tissue levels, exacerbated by environmental toxicants and unhealthy lifestyles [6].

A key objective in biological aging research is to identify biomarkers capable of predicting the biological age (B-age) of different tissues, as an alternative to relying solely on chronological age (C-age) [10,11]. The American Federation for Aging Research (AFAR) has devised a four-point set of criteria for biomarkers of aging as follows: (i) they must predict the rate of aging and life span better than C-age; (ii) they should be able to detect a basic process underlying the process of aging; (iii) they could be a non-invasive test like blood work or an imaging technique which can be repeated without causing much distress to the subject; and (iv) the technique can be used both for lab animals and humans and can be specific for a particular tissue or organ or reflect the biological age of the whole organism [12,13].The frequently used biomarkers to measure biological age are DNA methylation (DNAm), the length of telomere, transcriptomics, proteomics, and metabolomics [14,15]. The “DNAm clock” or “epigenetic clock” functions as an estimator of age built from epigenetic DNAm marks that are strongly correlated (r ≥ 0.8) with chronological age or time, and strongly associated with age-related phenotypes or outcomes [16]. B-age is estimated using biological clocks (B-clocks), a subset of which are epigenetic clocks [17]. B-clocks are the metrics of B-age based on DNAm patterns and reflects the health condition of an individual [18]. The difference between B-age and C-age is termed as B-age acceleration. Epidemiological investigations have linked B-age acceleration to a broad spectrum of pathologies, health conditions, lifestyles, mental states, and environmental factors. This implies that B-clocks encompass the significant biological processes associated with aging [16].

Therefore, to understand the underlying mechanisms of this complex aging process, this review focuses on epigenetic clocks and the potential causes of aging (environmental factors) that influence these clocks. The overall significance of this type of work is to identify preventable environmental factors that accelerate aging and to develop new therapies to enhance the quality of life by slowing or even reversing biological aging. We divide this review into two major sections—Section 2: Part 1. Biological clocks (B-clocks) as biomarkers of aging (first-generation, second-generation, third-generation, sperm clocks, single-cell clocks, RNA clocks, mitotic clocks, and pediatric clocks), and Section 3: Part 2. Identifying environmental drivers of aging using epigenetic clocks. We conclude with future needs to uncover and prevent environmental drivers of biological aging and disease.

## 2. Part 1. Biological Clocks as Biomarkers of Aging

### 2.1. Search Strategy and Selection Criteria

For Part 1, we have performed a systematic PubMed literature search using the keywords “Horvath’s clock”, “Hannum’s clock”, “mitotic clocks”, “RNA clocks”, “sperm epigenetic clocks”, and “pediatric clocks”. We completed the search using the PubMed database (https://www.ncbi.nlm.nih.gov/pubmed (accessed on 30 November 2023)) and Google scholar up to the end of November 2023. For Part 2, a Pubmed search was conducted separately with the keywords “epigenetic clocks and environmental exposures”, “epigenetic clocks and stress”, “epigenetic clocks and heavy metals”, “epigenetic clocks and air pollution”. We will not discuss epidemiological studies which showed no association of environmental contaminants with epigenetic aging. The selection of articles was made in compliance with the Preferred Reporting Items for Systematic reviews and Meta-Analyze (PRISMA) guidelines [19].

### 2.2. Epigenetic Clocks

Accumulating evidence suggests that there are plenty of connections between age and DNAm which led to the development of molecular epigenetic clocks representing biological age [20,21]. Two of the first molecular epigenetic clocks which are robust predictors of chronological age are Horvath [22] and Hannum [23] calculators which use DNAm data. Both demonstrate strong age correlations (r = 0.96 for Horvath and r = 0.91 for Hannum) and minimal mean deviations from chronological age (3.6 and 4.9 years, respectively) within their respective validation cohorts [22,23]. Aging is associated with a decrease in global DNAm along with an increase in local methylation at CpG islands and specific promoters [24,25,26,27]. Accelerated epigenetic aging is usually measured in DNA from whole blood and is defined as the difference between the B-age and C-age [28]. Accelerated B-age is associated with increased mortality and impaired physical and cognitive function [29]. The B-clocks based on DNAm are divided into (i) first-generation (G1) clocks (Hannum and Horvath), that are trained to predict C-age; (ii) second-generation (G2) clocks (PhenoAge, GrimAge), that have improved predictive power over G1 clocks and can predict longevity and the onset of age-related diseases; and (iii) third-generation clocks (G3) (DunedinPoAm), that are designed to estimate the pace of aging [18].

**Figure 1:**
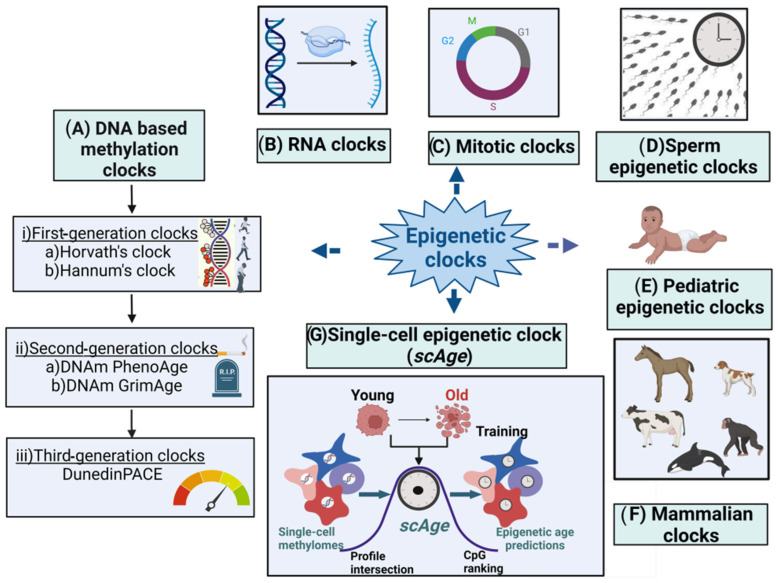
Major types of epigenetic clocks. (**A**) DNA-based biological clocks: (i) G1 clocks (e.g., Horvath’s and Hannum’s clocks); (ii) G2 clocks (e.g., DNAm PhenoAge and DNAm GrimAge); and (iii) G3 clocks (e.g., DunedinPACE). (**B**) RNA clocks. (**C**) Mitotic clocks. (**D**) Sperm epigenetic clocks. (**E**) Pediatric epigenetic clocks. (**F**) Mammalian clocks. (**G**) Single-cell epigenetic clock (scAge).

Many new B-clocks are being continuously developed till date—therefore in this article we present a snapshot of the widely used B-clocks as outlined in Figure 1 and Table 1. The article also discusses how the various environmental factors could cause accelerated epigenetic aging as measured by different epigenetic clocks.

### 2.3. First-Generation Molecular Epigenetic Clocks

#### 2.3.1. DNA Methylation-Based Molecular Epigenetic Clocks

The role of DNAm in the process of aging has been well established [45,46]. DNAm levels at a subset of loci strongly correlate with age and are applicable to a wide array of tissues, body fluids, and person’s age (from prenatal samples to centenarians) [22]. DNAm involves the addition of a methyl group to the 5′ position on cytosines in CpGs [47]. The methylation status of millions among the 28 million CpG dinucleotides in the human genome was observed to change as individuals age [48,49,50,51]. DNAm-based epigenetic clocks are generally built with a supervised machine learning method and can predict the age of the person or biological sample by combining the methylation values of tens to hundreds of selected CpGs [16]. The estimated age, known as B-age or DNAm age, is not only a reflection of C-age but also of the B-age of the DNA source. Biomarkers derived from DNAm provide mechanistic insights into health status, associating the pathogenesis of a disease with biological aging [52].

Age acceleration is characterized by variances between age estimates derived from DNAm and chronological age C-age. Positive age acceleration indicates that the DNAm-based age is older than the chronological age, while negative age acceleration signifies a DNAm-based age that is younger than the chronological age. Age acceleration serves as the central focus for all clocks, and we discuss the most commonly utilized as well as emerging clocks in the following sections.

#### 2.3.2. Horvath’s Clock

In 2011, a UCLA research team published the first single-tissue DNAm-based age estimator using DNA extracted from saliva [53]. The following year, an Italian group discovered using blood a new epigenetic marker of age based on a single CpG [54]. In 2013, Horvath and collaborators pioneered the initial multi-tissue predictor of age through the examination of approximately 8000 samples derived from 51 healthy human tissues, including various cell types such as liver, kidney, immune, and brain cells, alongside around 6000 cancer samples. Their findings demonstrated that DNA methylation (DNAm) can serve as a dependable age indicator in normal/non-cancerous tissues [22]. Horvath’s DNAm age is calculated based on methylation measurements at 353 loci, present on Illumina’s 450 K and 27 K DNAm microarrays [22]. The clock showed remarkable accuracy, predicting age with a correlation of 0.96 and a median error of 3.6 years across various tissues and cells [22]. Horvath’s clock stands out for its correlation with C-age across various tissue types, applicability to pediatric samples, strong correlation with gestational age (differentiation day), and the consistency of its age estimates across multiple tissues [55].

A recently introduced DNA methylation array, the HorvathMammalMethylChip40, was designed to analyze CpGs that exhibit high conservation across mammals. This array surveys 37,000 CpGs, offering a platform for investigating epigenetic aging in mammals [56]. Utilizing this array, researchers have created multi-tissue universal clocks that can be applied to various mammalian species [56,57]. Using this array, epigenetic clocks for naked mole rats [58], elephants [59], marsupials [60], cats [61], pigs [62], livestock species (e.g., goat, cattle, sheep, and deer) [63], and horses [64] were developed. The B-clocks for mice and other mammals were developed using samples from blood and other tissues such as adipose, heart, kidney, liver, lung, spleen, muscle, etc. [65]. These readily available B-clocks can be used for wildlife conservation purposes and in breeding programs.

#### 2.3.3. Hannum’s Clock

In a nearly simultaneous investigation in 2013, Hannum and collaborators constructed a quantitative aging model based on measurements taken from over 450,000 CpG markers in the whole blood of 656 individuals aged 19 to 101 [23]. The Hannum clock selectively utilizes 71 CpG sites sourced from the Illumina 450 k array, strategically positioned near genes linked to aging. This targeted selection contributes to its high accuracy in predicting age. The clock has undergone validation in human blood, and ongoing initiatives are focused on adapting the clock for implementation in model organisms like mice [66]. Hannum’s age estimator is designed for adult blood samples, resulting in skewed estimates when applied to children and non-blood tissues [23,67,68]. Furthermore, it is affected by age-related changes in blood composition [5,69].

### 2.4. Second-Generation Epigenetic Clocks

#### 2.4.1. DNAm PhenoAge

Hannum and colleagues’ blood-based algorithm, along with Horvath and colleagues’ multi-tissue algorithm, generates biological age estimates (DNAm age) that demonstrate a strong correlation with chronological age (C-age), well exceeding r = 0.90 across samples spanning the entire age range. Nevertheless, these biological age estimators display statistically significant associations with numerous age-related health conditions [30]. Consequently, Levine et al. developed a DNA methylation-based biomarker referred to as DNAm PhenoAge. This biomarker was designed to predict a surrogate measure of “phenotypic age”, distinguishing between morbidity and mortality risks among individuals of the same chronological age [30]. It was devised by calculating a weighted average of 10 clinical characteristics: body mass index (BMI), chronological age (C-age), albumin, creatinine, glucose, and C-reactive protein levels, lymphocyte percentage, cell volume, alkaline phosphatase, and blood cell counts. Subsequently, these values were modeled against DNA methylation (DNAm) levels in blood using a penalized regression model. This approach led to the automatic selection of 513 CpGs, the weighted average of which serves as an effective estimate of phenotypic age. This method represents a significant advancement over first-generation DNA methylation biomarkers (Hannum and Horvath) in predicting morbidity [30].

#### 2.4.2. DNAm-Based Biomarker of Mortality (DNAm GrimAge)

In 2019, DNAm GrimAge, an EC based on estimations of plasma protein levels that can predict lifespan and health span was developed by Lu et al. [31]. A unique two-stage method was utilized to create DNAm GrimAge. In the initial stage, surrogate DNA methylation biomarkers associated with physiological risk or stress factors were identified. These biomarkers encompass specific plasma proteins: adrenomedullin, C-reactive protein, plasminogen activation inhibitor 1 (PAI-1), and growth differentiation factor 15 (GDF15) [70,71]. Smoking is a pivotal risk factor for both mortality and morbidity. Consequently, the authors employed a DNA methylation-based estimator of smoking pack-years. In the second stage, these biomarkers were amalgamated into a unified composite biomarker of lifespan known as DNAm GrimAge, expressed in units of years.

A comprehensive meta-analysis, incorporating over 7000 Illumina array measurements, substantiated that DNAm GrimAge surpasses other DNA methylation-based predictors as a more accurate predictor of lifespan. DNAm GrimAge is a linear combination of C-age, sex, and DNA methylation-based surrogate biomarkers for seven plasma proteins and smoking pack-years. It outperforms all other DNA methylation-based biomarkers across a range of health-related metrics [31]. The G2 clocks, including DNAm PhenoAge and DNAm GrimAge, surpass current molecular biomarkers of aging due to their robust correlation not only with C-age but also with various age-related conditions [15].

### 2.5. Third-Generation Epigenetic Clocks

A third-generation epigenetic clock, DunedinPACE (Pace of Aging Calculated from the Epigenome), was developed by training on an extensive set of direct biomarkers related to health and diseases. These include BMI, waist-hip ratio, glycated hemoglobin (HbA1c), leptin, blood pressure, lung function, cognitive function, grip strength, and motor coordination, among others. Belsky et al. introduced it in 2021 as an innovative blood biomarker for assessing the pace of aging in the fields of gerontology and geroscience [72]. The researchers utilized data from a birth cohort (*n* = 1037 babies) in Dunedin, New Zealand, born during 1972–1973, tracking them through midlife. Aging was observed to lead to a gradual and progressive deterioration that affected various organ systems. To capture these changes, the authors examined longitudinal shifts in 19 biomarkers, assessing cardiovascular, metabolic, renal, hepatic, immune, dental, and pulmonary systems at ages 26, 32, 38, and 45 years. Analyzing this dataset resulted in the creation of a metric called the “Pace of Aging”, quantifying each participant’s individual rate of biological aging. DunedinPACE demonstrated high test–retest reliability, exhibited associations with morbidity and mortality, and identified accelerated aging in young adults who had experienced childhood adversity [72].

DunedinPACE differs from other DNA methylation clocks in both its development methodology and interpretation [72]. DunedinPACE contributed additional predictive value beyond clocks like Horvath, Hannum, PhenoAge, or GrimAge in analyzing the occurrence of new cases of morbidity, disability, and mortality. While other clocks estimate aging progression up to a specific point in time, DunedinPACE offers DNAm estimates of the Pace of Aging, representing the ongoing rate of decline in system integrity.

### 2.6. Mitotic Clocks

The number of cell divisions of a given tissue type in an individual is of great interest since it allows the study of biological aging using a new molecular target (mitotic age) and can predict prospective cancer risk in individuals [73]. DNAm-based mitotic-like clocks can keep count of the cumulative number of DNAm errors arising during cell division [33,74]. There have been three epigenetic mitotic clocks reported in the literature to date: (a) Epigenetic Time to Cancer (EpiTOC), (b) Epigenetic Time to Cancer-2 (EpiTOC2), and (c) Mitotic Age (MiAge) [33,34,73]. In 2016, Yang et al. developed a DNA methylation-based age-correlated model named “EpiTOC” (Epigenetic Timer of Cancer). This model operates as a mitotic clock in both normal and cancerous tissues [33]. It utilizes CpGs located in gene promoters associated with the PRC2 polycomb repressive complex, also known as Polycomb group targets (PCGTs), in human embryonic stem cells (hESCs). These CpGs remain constitutively unmethylated in a ground state of age zero across various fetal tissue types. As chronological age (C-age) increases, DNAm levels also rise, enabling researchers to evaluate variations in DNA methylation in both aged and oncogenic tissues.

### 2.7. Sperm Epigenetic Clocks

Studies have shown that sperm DNAm mediated the age-related effects of male C-age on poor reproductive outcomes like fertilization rates, embryo development, and live birth [75]. However, somatic tissue-based clocks cannot precisely predict epigenetic aging in germ cells [22]. To address this constraint, Jenkins et al. in 2018 developed the inaugural sperm-specific epigenetic clock within a diverse population, encompassing sperm donors, infertile men, and the community. This was achieved by employing regional methylation levels from the Illumina 450 K array [76]. In 2022, Pilsner et al. undertook a more thorough examination, employing the Illumina EPIC array to formulate a novel CpG-based sperm epigenetic age (SEA) within a cohort of 379 participants with the common goal of conception [35]. These results indicate that the sperm epigenetic clock could serve as a novel biomarker for the reproductive success of couples. Both the study conducted by Jenkins et al. and that of Pilsner et al. demonstrate that sperm epigenetic age (SEA) is influenced by environmental factors, as evidenced by accelerated SEA observed among smokers in clinical cohorts, sperm donors, and the general public.

### 2.8. Single-Cell Epigenetic Clock Framework (scAge)

Most existing B-clocks rely on measurements derived from samples containing many cells [77]. Bulk samples are valuable for identifying average methylation patterns in tissues, but they neglect the epigenetic heterogeneity that might exist among individual cells [77,78]. To address this concern, the scAge, an epigenetic clock framework, was devised to allow the profiling of biological age at the level of individual cells [38]. Due to the variable coverage of CpGs between cells, the authors employed a ranked intersection algorithm that is agnostic to which CpGs are covered in each cell. This method synthesizes the C-age of tissues while revealing the inherent epigenetic heterogeneity among individual cells. The authors successfully utilized the scAge model to monitor aging in hepatocytes and embryonic fibroblasts, demonstrated reduced epigenetic aging in muscle stem cells, and tracked age dynamics in embryonic stem cells. Consequently, the scAge framework can serve as a valuable tool for exploring epigenetic aging trajectories at single-cell resolution, offering exciting applications in emerging single-cell technologies.

### 2.9. RNA Clocks

Changes in gene expression in addition to DNAm have also been related with age-related diseases [79,80,81,82,83,84,85]. Numerous computer programs exist for predicting DNA methylation (DNAm) age from human DNAm data assessed on the Illumina Infinium HumanMethylation450K BeadChip, such as RNAAgeCalc. However, when it comes to gene expression data, age-related signatures have been developed using either non-human sources or very limited tissue samples [36].

#### 2.9.1. RNAAgeCalc: A Multi-Tissue Transcriptional Age Calculator

RNAAgeCalc stands out as the initial versatile transcriptional age calculator that works across tissues and provides tissue-specific predictions. Its development involved utilizing RNA-Seq data from the Genotype-Tissue Expression (GTEx) Program [86]. Utilizing publicly available GTEx data, a database that encompasses genes across various tissues, the transcriptional age calculator RNAAgeCalc, was constructed with tissue-specific capabilities [36]. The authors subsequently verified, using the calculator, that transcriptional age acceleration was associated with reduced mortality risk and mutation burden in TCGA cancer samples. This provided corresponding insights in comparison to DNAm age [36]. Therefore, RNAAgeCalc may advance our efforts for novel treatments for age-related diseases.

#### 2.9.2. Multi-Tissue RNA Clock (MultiTIMER)

It was developed by combining previous data about gene-function associations from the Molecular Biology of the Cell Ontology (MBotC) [87] with a machine learning approach to detect the cellular processes and their associated genes which are predictive of age [37]. MultiTIMER determines the biological age (B-age) of cells based on their transcriptional profiles, assessing critical cellular processes. Applied to over 70,000 transcriptional profiles, MultiTIMER revealed that the aging process correlates with distinct aging phenotypes and responds to cellular stressors and interventions. In comparison to RNAAgeCalc, which predicts cell age from a set of 1600 genes expressed differentially across tissues during aging, MultiTIMER is more robust [36].

### 2.10. Pediatric Epigenetic Clocks

B-age acceleration measures developed using adult and pediatric samples are not suitable for estimating gestational age (G-age) and are inappropriate for use in neonates. These B-clocks are not trained on G-age data and do not exhibit correlation with G-age. In recent years, various clocks have been specifically designed for predicting G-age and pediatric C-age in pediatric populations [88]. Widely used G-age clocks include the Knight clock which was developed from cord blood data using both 27 K and 450 K arrays and consists of 148 CpGs [39], and the Bohlin clock which consists of 96 CpGs and was developed using cord blood data from 450 K data [40]. The Lee clock was designed using placental data from the 450 K and EPIC arrays and is based on 558 CpGs [41]. The Mayne clock was developed using placental data from both 27 K and 450 K arrays and consists of 62 CpGs [43]. The NeoAge clock was developed to forecast both postmenstrual age (PMA, time from estimated conception onward) and post-natal age (PNA, time elapsed after birth) for preterm infants. This was achieved using buccal cell samples and considering 303–522 CpGs [44]. As far as pediatric clocks are concerned, the Pediatric-Buccal-Epigenetic (PedBE) clock [42], comprising 94 CpGs, primarily focuses on the pediatric population (0–20 years old). The PedBE clock was trained in buccal cell samples using the platform of 450 K and EPIC arrays; it is a better predictor when saliva or buccal cells are used compared to blood DNA. The Horvath clock is also an accurate age predictor in children along with adults [22].

### 2.11. Nutrition and Epigenetic Clocks

Exploring nutritional interventions emerges as a promising approach to promote healthy aging, given the growing body of evidence indicating their potential to positively impact the health status of individuals [89,90]. Key modifiers of epigenetic patterns include dietary factors that supply S-adenosyl-methionine for one-carbon metabolism and polyphenols, such as flavanols, which can inhibit the activity of DNA methyltransferases (DNMTs) [91]. Intervention trials were conducted in 44 participants with folic acid + vitamin B12 (GSE74548) and in 13 participants with monomeric and oligomeric flavanols (MOF) (GSE54690) [91]. DNAm patterns were evaluated in publicly available Illumina Infinium 450 K methylation datasets. Following supplementation with folic acid + vitamin B12, global DNAm levels were observed to increase in unmethylated regions like CpG islands and shores, while decreasing in highly methylated regions after intervention with MOF. In women with the methylenetetrahydrofolate reductase (MTHFR) 677CC genotype, supplementation with folic acid + vitamin B12 led to a reduction in B-age, as estimated by the Horvath “epigenetic clock” model. Additionally, a population-based prospective cohort study involving 1346 newborns, utilizing Bohlin’s and Knight’s clocks, demonstrated an association between higher maternal plasma homocysteine concentrations and positive G-age acceleration, suggesting a faster epigenetic aging rate compared to clinical gestational aging [92]. However, cord serum vitamin B12 concentrations were associated with negative G-age acceleration, indicating slower epigenetic than clinical gestational aging [92].

The Mediterranean diet is recognized for its well-balanced combination of nutrients, antioxidants, and anti-inflammatory compounds, and it has been proposed as a potential preventive measure against telomere shortening [93,94]. A pilot intervention study, involving 120 elderly healthy subjects (60 Italians, 60 Poles), was designed to explore the effects of a Mediterranean-like diet over one year. The diet was specifically tailored to meet the nutritional requirements of individuals over 65 years of age. The study aimed to assess the impact on age-related diseases and functional decline by measuring changes in their biological age (B-age) using Horvath’s clock [95]. Polish females and individuals who exhibited higher epigenetic age at baseline appeared to derive greater benefits from the Mediterranean-like diet. Additional research is needed to understand why certain individuals respond more favorably to specific interventions. This is a crucial step in the development of personalized nutritional interventions for precision medicine-based anti-aging strategies.

A 24-month study involving 219 women from the “Diet, Physical Activity, and Mammography” (DAMA) study in Florence, Italy, explored the potential favorable effects of enhanced dietary habits and increased physical activity on the development of breast cancer and aging biomarkers in healthy postmenopausal women aged 50–69 years. [96]. DNAm was assessed both at the study’s outset and upon completion. Women engaged in the dietary intervention exhibited a noteworthy deceleration of the DNAm GrimAge clock. Concurrently, increased physical activity resulted in a significant reduction of stochastic epigenetic mutations in critical cancer-related pathways. The DAMA study employed non-extreme interventions, easily achievable through lifestyle modifications. This suggests that policy intervention programs promoting a healthy diet and physical activity could substantially reduce the societal burden of aging-related pathological conditions and diseases. In conclusion, diverse anti-aging strategies, including stress reduction, experimental drugs, and nutritional interventions, can be adopted to decelerate epigenetic aging.

## 3. Part 2. Environmental Toxicants Affecting Epigenetic Clocks

**Figure 2:**
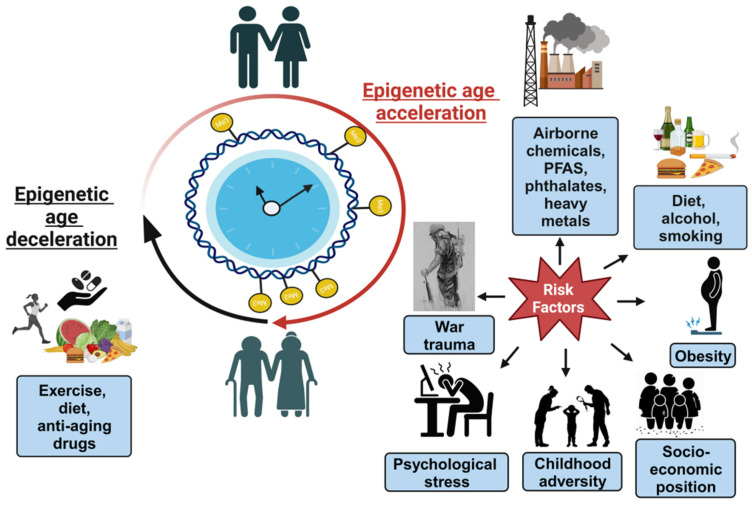
Environmental agents affecting epigenetic clocks. Factors causing biological age (B-age) acceleration: chemical agents (e.g., airborne chemicals, phthalates, PFAS, and heavy metals); lifestyle factors (e.g., diet, alcohol, smoking, and obesity); and psychological factors (e.g., war trauma, childhood adversity, stress, socio-economic status, etc.). B-age acceleration could be improved with a healthy diet, physical activity, and anti-aging drugs like rapamycin, etc.

Several lifestyle, biological, social, and environmental factors might accelerate biological aging, and their associations with epigenetic clocks have been investigated as outlined in Figure 2 [97,98,99]. Studies indicate that the acceleration of epigenetic clocks was significantly associated with socio-economic status, air pollution, BMI, HIV infection, alcohol use, and male sex [100]. Here, we wanted to summarize existing research findings and provide a snapshot of the various environmental, social, and biological factors associated with epigenetic aging.

### 3.1. Airborne Chemicals

Air pollution is a major environmental hazard for human health [101]. As per a 2016 report from the World Health Organization (WHO), 92% of the global population is exposed to air pollution levels exceeding permissible limits [102]. Air pollution encompasses various components, including particulate matter (PM), ozone (O_3_), sulfur dioxide (SO_2_), nitrogen oxides (NO_x_), carbon monoxide (CO), benzene, black carbon, polycyclic aromatic hydrocarbons, and toxic metals [103]. Several studies have indicated that exposure to air pollution on a long-term basis is responsible for accelerated epigenetic aging [104,105]. Upon inhalation, airborne particulate matter (PM) is deposited across the respiratory tract, contributing to inflammation and damaging the airway epithelium [106,107]. PM10 and PM2.5, referring to particulate matter with aerodynamic diameters of <10 μm and <2.5 μm, respectively, constitute key elements of particulate air pollution. The composition of these particles varies significantly based on geographic regions [108]. Studies have demonstrated that PM10 can impact the methylation of genes that regulate the circadian cycle [109,110].

In a panel study conducted in China, 76 healthy adults aged 60–69 years were recruited to investigate whether exposure to PM2.5 is a significant risk factor for cardiovascular aging and the development of other comorbidities [111]. The study compared the effectiveness of seven types of B-age and C-age to assess the impact of short-term PM2.5 exposure on blood pressure, a clinical marker for cardiovascular aging. Utilizing both G2 indicators (PhenoAge and SkinBlood Clock) and a G3 B-age indicator (DunedinPoAm), it was observed that changes in blood pressure were significantly and positively influenced by PM2.5 mass exposure. Consequently, B-age could serve as a predictive measure for cardiovascular susceptibility to air pollution exposure in this population.

Laan et al. conducted a study in China involving factory workers (*n* = 50) to explore the potential association between occupational air exposure to chemical carcinogens (e.g., benzene, trichloroethylene (TCE), or formaldehyde) and epigenetic aging [112]. In all three studies, DNAm was assessed using the HumanMethylation450 BeadChips (Illumina HM450K arrays). The authors employed five different epigenetic clocks, namely, Horvath, Skin-Blood Clock, Hannum, PhenoAge, and GrimAge. The findings revealed that exposure to benzene accelerated the Hannum and Skin-Blood B-clocks, and it was associated with shorter DNAm estimates of telomeric length, which provides a more accurate measure of cell replication than actual telomere length. In the TCE study, an acceleration in B-age was observed among workers exposed to <10 ppm of TCE compared to controls, particularly for the Skin-Blood Clock. Overall, the study provided evidence that exposures to benzene and TCE can impact biomarkers of epigenetic aging.

A study conducted with the Scotland-based Lothian Birth Cohort 1936 aimed to assess the sensitive periods during an individual’s life course when air pollution would exert a more pronounced impact on DNAm-based markers of biological aging [52,113]. The study involved the recruitment of 525 individuals, including both men and women, with a total of 1782 observations. Blood samples were collected between the ages of 70 and 80 years, and Horvath, Hannum, PhenoAge, GrimAge, and DNAmTL were utilized for the analysis. The participants’ residential history was linked to annual levels of a mixture of air pollutants, including fine particles (PM2.5), sulphur dioxide (SO_2_), nitrogen dioxide (NO_2_), and ozone (O_3_). Exposure to air pollution during young-to-middle adulthood was associated with biological age (B-age) measured using Horvath’s epigenetic clock. Shorter estimated telomere lengths were observed among males with higher exposure to air pollution in mid-adulthood, indicating an accumulating impact of air pollution across the life course. In females, air pollution specifically around birth was associated with estimated telomere attrition. This study highlights the role of DNAmTL as a biomarker of cellular aging susceptible to the effects of air pollution exposure.

A separate study, encompassing six longitudinal population-based birth cohorts in diverse populations (UK, France, Spain, Lithuania, Norway, and Greece), was conducted. The findings revealed that exposure to tobacco smoke during pregnancy/childhood and indoor particulate matter exposure during childhood are linked to accelerated epigenetic aging in children [114]. The study recruited 1173 children, approximately 7 years old. Age acceleration was assessed using the Skin-Blood clock, utilizing child blood DNA measured using Infinium 450 K. A notable strength of the study lies in its examination of two distinct time periods—pregnancy and childhood—recognized as critical stages of vulnerability in a child’s development [115]. Aging has emerged as a global public health concern, emphasizing the need for new studies in child populations. Such research can aid government agencies in formulating policies to reduce environmental exposures and promote “healthy aging” from early life.

A study was conducted to investigate the connection between prenatal exposure to air pollution and epigenetic aging at birth. This research involved two pregnancy cohorts that included women with a previous child diagnosed with autism spectrum disorder (*n* = 332 mother–child pairs) [116]. In the analysis of cord blood tissue, DNAm age was computed using the Knight and Bohlin epigenetic clock algorithms. The findings indicated that exposure to O_3_, PM2.5, and PM10 during both the preconception period and pregnancy led to a deceleration of biological age (B-age) at birth. The authors further identified that the early- to mid-pregnancy phase was a critical period for the association between particulate matter (PM) and epigenetic aging. This study suggests that prenatal environmental stress may influence fetal programming, as reflected by alterations in epigenetic aging.

### 3.2. Phthalates

Phthalates act as endocrine-disrupting chemicals, impair reproductive health, and cause asthma, rheumatoid arthritis, cardiovascular disease, and more [117,118,119,120]. A study conducted in China showed that airborne chemicals including phthalates significantly increased the acceleration of the epigenetic biomarkers for phenotypic age [121]. The study recruited 76 healthy, non-smoking older adults aged 60–69 years. DNAm levels were measured in blood samples and used to calculate epigenetic aging biomarkers including Hannum clock, Horvath clock, PhenoAge, GrimAge, and a DNAm estimator of telomere length (DNAmTL). The study revealed that phthalates accelerated PhenoAge. These findings might enable the designing of more targeted disease prevention strategies to mitigate the toxic effects of environmental chemicals.

In 2022, Pilsner et al. conducted a study examining the correlation between urinary phthalate metabolites and sperm epigenetic age (SEA) in male partners of couples intending to start a family [122]. In the study involving male partners (*n* = 333), 11 urinary phthalate metabolites were measured. In the multivariate analyses focusing on individual metabolites, nine of them exhibited positive trends with sperm epigenetic age (SEA) in the range of 0.05–0.47 years. Phthalate metabolites have been associated with changes in sperm methylation [123] which indicate that continuous exposure to phthalates might interfere with the activities of DNAm machinery of spermatogonia stem cells during spermatogenesis and cause epigenetic modifications over time [124]. The methylation defects that accumulate in spermatogonia are subsequently transmitted during sperm maturation, and Pilsner’s sperm epigenetic clock can quantify these changes in sperm [35]. Men with elevated phthalate exposures may bear sperm populations displaying epigenetic signatures associated with aging. These signatures could potentially contribute to infertility or other adverse reproductive outcomes.

### 3.3. Heavy Metals

Iron is an essential nutrient for humans and too little or too much can be harmful to health [125]. Several studies have reported that excess iron accumulation accelerates human aging [126,127,128]. In a comprehensive genome-wide association study (GWAS), the association between four systemic iron status biomarkers (ferritin, serum iron, transferrin, and transferrin saturation) and four biomarkers for biological age (GrimAge, PhenoAge, intrinsic biological age acceleration, and Hannum) was investigated on a large scale [129]. Genetic correlations were assessed through linkage disequilibrium score (LDSC) regression, Mendelian randomization (MR), and MR based on Bayesian model averaging. The LDSC results revealed genetic correlations between serum iron and PhenoAge, as well as transferrin saturation and PhenoAge. Furthermore, the study identified that ferritin and transferrin saturation significantly elevated all four measures of biological age. In summary, this research emphasizes the genetic causal relationship between systemic iron status and biological age acceleration.

A population-based study was undertaken in American Indian communities located in Arizona, Oklahoma, and North and South Dakota (*n* = 2301) to clarify the connection between metal exposures and biological age acceleration [130]. Compared to other US populations, this cohort is exposed to higher levels of arsenic (As), cadmium (Cd), and tungsten (W) [131]. They also have hyperglycemia, and the high burden of type 2 diabetes increases urinary Zn excretion [132]. Blood methylation data from participants were utilized to compute age acceleration using various biological clocks (PhenoAge, GrimAge, DunedinPACE, Hannum, and Horvath). The levels of urinary metals (arsenic (As), cadmium (Cd), tungsten (W), zinc (Zn), selenium (Se), and molybdenum (Mo)) were adjusted for creatinine and categorized into quartiles. The results revealed that the combination of nonessential metals (W, As, and Cd) was positively associated with increased GrimAge acceleration and DunedinPACE, while the essential metal mixture (Se, Zn, and Mo) was linked to lower biological age acceleration. The most robust associations were observed between Cd and DunedinPACE and GrimAge acceleration. Therefore, investigating the impact of metal mixtures on biological age acceleration is crucial for understanding the biological processes underlying various age-related health conditions.

A study was undertaken to explore the impact of serum lead (Pb), mercury (Hg), manganese (Mn), and copper (Cu) on DNA methylation (DNAm) age, employing three DNAm-based biomarkers of aging (Horvath Age, PhenoAge, and GrimAge) [133]. The longitudinal data were collected from adults (*n* = 290, mean age: 51 ± 17) residing in an environmentally polluted post-industrial city (Detroit, Michigan) from 2008 to 2013. Lead (Pb) showed a positive association with GrimAge acceleration, and mercury (Hg) exhibited a positive association with PhenoAge acceleration. However, consistently negative associations were observed between manganese (Mn) and PhenoAge acceleration, as well as Hg and Horvath age acceleration. Copper (Cu) displayed a strong U-shaped relationship with both PhenoAge and GrimAge acceleration. An increase in total exposure to the observed mixture of metals resulted in increased PhenoAge and GrimAge acceleration and decreased Horvath age acceleration. These findings suggest that an elevation in serum Pb or Hg from the 25th to 75th percentile is linked to approximately a 0.25-year increase in two epigenetic markers of all-cause mortality in a population of adults in Detroit, Michigan. Therefore, population-wide exposure to toxic metals (e.g., Pb and Hg) at low levels can contribute to accelerated biological aging.

### 3.4. Per- and Polyfluoroalkyl Substances

Per- and polyfluoroalkyl substances (PFAS) are a group of widespread man-made persistent organic pollutants with highly stable perfluorinated carbon chains. PFAS are ubiquitous in households, workplaces, and the environment [134,135,136]. Exposure to per- and polyfluoroalkyl substances (PFAS) during in utero development has been associated with alterations in offspring DNA methylation (DNAm) at both individual CpG sites and regions within genes that play roles in growth, cardiometabolic, and immune health [137,138].

A pilot study was conducted at Ronneby, Sweden, which had a highly PFAS-contaminated drinking water supply (primarily PFOS and PFHxS), from the mid-1980s to December 2013 [139]. The study was conducted to elucidate whether PFAS exposure is associated with an alteration of DNAm and B-age acceleration among women [139]. A total of 59 women (aged 20–47 years) were recruited from the Ronneby Biomarker Cohort, while the control group (*n* = 226, aged 5–59 years) was recruited from Karlshamn, a nearby municipality with uncontaminated drinking water supply and similar socioeconomic status. The average serum per- and polyfluoroalkyl substance (PFAS) levels for different PFAS compounds in the Ronneby cohort were approximately 10–100 times higher than the control group. The Infinium MethylationEPIC BeadChip was employed to analyze the genome-wide methylation of whole-blood DNA. Although PFAS exposure was associated with methylation changes at specific sites and regions, there was no observed association between PFAS exposure and biological age acceleration according to the Skin-Blood clock.

Another exploratory study was designed to evaluate how PFASs might influence DNAm and B-age acceleration among school-age children (*n*= 63, aged 7–11 years) from the Ronneby Biomarker Cohort (Sweden) [140]. The control group had exposure at background levels (*n* = 32; perfluorooctane sulfonic acid: median 2.8 and range 1–5 ng/mL) and the exposed group was exposed to very high levels of PFASs (*n* = 31; perfluorooctane sulfonic acid: median 295 and range 190–464 ng/mL). The two groups were similar with respect to sex, age, and BMI. The genome-wide methylation of whole-blood DNA was analyzed using the Infinium MethylationEPIC BeadChip kit. While associations between PFAS with CpG sites and regions were reported, there were no differences in B-age acceleration between the high and low PFAS exposure group.

A study was conducted with incumbent firefighters (*n* = 197) from three cities in the USA to assess whether PFAS serum concentrations were associated with EA biomarkers [141]. The serum concentrations of nine PFASs, blood leukocyte DNAm, and B-age indicators via the EPIC array were quantified. Multiple epigenetic clocks, such as Horvath, Hannum, Skin-Blood, PhenoAge, and GrimAge, were used for the study. The authors also calculated IEAA and EEAA. Perfluorohexane sulfonate (PFHxS) was positively associated with three clocks (EEAA, Hannum, and Skin-Blood), linear perfluorooctanoate (N-PFOA) with six clocks (all except GrimAge), and the sum of perfluoromethylheptane sulfonate isomers (Sm-PFOS) with two clocks (IEAA and Horvath). There were no statistically significant associations between the other PFAS, except for inverse associations between perfluorodecanoate (PFDA) and perfluoroundecanoate (PFUnDA) with GrimAge (*p <* 0.05).

### 3.5. Psychosocial and Lifestyle Factors Affecting Epigenetic Clocks

Psychological stress has long been associated with affecting human longevity on a molecular level by causing oxidative stress and telomere attrition [142]. A growing number of studies show that psychiatric symptoms, such as major depression [143], bipolar disorder [144], posttraumatic stress disorder (PTSD) [145,146,147,148,149], and internalizing symptoms in children [150], may accelerate cellular aging in the epigenome.

A study using the Hannum clock was conducted on a cohort of young trauma-exposed military veterans (median age = 32 years), which showed that lifetime PTSD severity was associated with accelerated DNAm age [145]. The same group replicated the previous study design with a group of mid-aged veterans who were approximately 20 years older than the previous study with a mean age of 52.58 years [151]. The sample comprised of white, non-Hispanic veterans (295 (87.0%) men and 44 (13.0%) women). The study demonstrated that PTSD hyperarousal symptoms were associated with accelerated DNAm age which gave rise to an approximately 13% increased risk for mortality over the course of approximately 6.5 years.

Another study involving 179 Iraq/Afghanistan war veterans was conducted to examine whether PTSD, depression, generalized anxiety, and alcohol-use disorders were related with accelerated B-age over time using Horvath and Hannum’s clocks [152]. The results indicated that alcohol-use disorders and PTSD were associated with an accelerated B-age over time.

A study was conducted in 2018 by Wolf et al., which was one of the largest and demographically diverse studies elucidating the associations between trauma exposure, PTSD, and accelerated DNAm aging. It consisted of nine studies and over 2000 participants from the Psychiatric Genomics Consortium for PTSD [153]. The study showed that both trauma exposure as a child and PTSD severity during lifetime could give rise to accelerated epigenetic aging. A significant observation from the study was that it was not lifetime but only childhood trauma exposure that affected aging. Therefore, there could be two possibilities—(a) there are important windows during childhood when the effects of trauma exposure have most pronounced effects; and/or (b) childhood trauma could lead to a longer period of psychiatric distress which causes a greater burden. The authors also observed a negative association between the population of CD4 T cells and both DNAm age residuals and a positive association between NK cells and Hannum age residuals. These findings indicate that immune cells and inflammation might play a role in affecting the pace of E-aging.

A study elucidated longitudinal changes in B-age before (t0) and after (t1) exposure to work-related trauma among Australian University paramedicine students (*n* = 40) during a 12 month-period [154]. The study further evaluated whether psychological risk factors (psychological distress, PTSD symptoms, and professional life) and protective factors (social support and organizational membership) can influence E-aging at both time points. All participants were documented to experience some form of trauma (e.g., patient death, witnessing suicide scenes, or attending aggressive patients) between baseline and follow-up [154]. The Horvath clock, GrimAge, and the Skin-Blood clock were used for the analyses. Baseline E-age and follow-up B-age were positively associated with risk factors of psychological distress and PTSD symptom severity. Students who were part of a psychological support group at the start of the paramedicine course had significantly reduced GrimAge acceleration at baseline and post-trauma exposure. In conclusion, the results indicate that E-aging is dynamic, and protective factors may influence E-aging at various time-points.

A study recruited a healthy young- to-middle-aged community population (*n* = 444, aged 18–50) and observed that cumulative stress was positively related with accelerated GrimAge and other stress-related physiologic measures such as adrenal sensitivity and insulin resistance [155]. The association between stress and age acceleration was most evident in those participants with specific psychological traits, such as poor emotion regulation and self-control, and was further modulated by lifestyle factors such as smoking and BMI.

### 3.6. Obesity and Its Effect on Epigenetic Clocks

Obesity has reached global epidemic proportions (WHO European Regional Obesity Report 2022). Obesity predisposes an individual to several age-related disorders such as cardiovascular disease (CVD), hypertension, type 2 diabetes mellitus (T2DM), and cancer [156]. In general, obesity increases the risk of premature death by 1.45- to 2.76-fold and reduces lifespan by up to 20 years [157].

A study was conducted on severely obese individuals to investigate the effect of bariatric surgery and subsequent weight loss on DNAm and biological age estimated using Horvath’s clock [158]. A cohort of 40 severely obese individuals (65% being women) with a mean age = 45.1 ± 8.1 years were examined at the time of surgery and at three follow-up visits, i.e., 3, 6, and 12 months after surgery [158]. The mean B-age before surgery was 3.17 years higher compared to the C- age, while the mean B-age post surgery was 2.26 years higher compared to the C-age. B-age acceleration was smaller after surgery (mean = − 0.92, *p* value = 0.039), suggesting a significant improvement of biological age.

### 3.7. Psychosocial Factors Affecting Newborn Epigenetic Aging

E-aging in early life occurs at a different temporal pace as compared to older age, and involves different DNAm patterns [159]. A study in the Democratic Republic of the Congo sought to identify the association between four measures of maternal stress, namely, general trauma, sexual trauma, war trauma, and chronic stress, with changes in DNAm in mothers and newborns [160]. The study recruited 155 mother–newborn dyads (mean maternal age: 26.2 years) from the general population as well as victims of sexual violence. Horvath’s clock, IEAA, EEAA, DNAmTL, PhenoAge, and GrimAge were used to test the relationships between biological aging and maternal stress in mothers and newborns. The sexual trauma of mothers gave rise to accelerated B-age across several B-clocks. General trauma and war trauma were also associated with newborn B-age acceleration using the EEAA.

Another study was aimed to elucidate the association between early life trauma, pubertal timing, and four epigenetic measures of B-age acceleration—Horvath, Hannum, PhenoAge, and GrimAge—were used for the study [161]. The participants were premenopausal mothers (*n* = 183, mean age 42 years) of children between 2 and 16 years of age. Participants were divided into two groups—caregiver group (*n* = 92, mothers of a child with a diagnosis of autism spectrum disorder) and control group (*n* = 91, mothers of developmentally normal children). Secondary analyses investigated the effects of types of childhood maltreatment (e.g., abuse, neglect) and chronic stress status (e.g., caregiver of an autistic child versus non-caregiver). It was found that compared to controls, caregivers had higher levels of early life abuse, neglect, and trauma. Using GrimAge, authors found that early life abuse and menarche were both associated with faster B-age acceleration in adulthood. However, chronic stress status did not influence the results.

In a clinical study conducted in Berlin, Germany, the Pediatric-Buccal-Epigenetic (PedBE) tool was employed to predict DNA methylation (DNAm)-based aging deviation in children aged 3–5 years, both with and without internalizing disorders. The study also aimed to assess the moderating effect of maltreatment exposure. The total sample size included 158 children: 49 with current internalizing disorders and 109 without. Within the total sample, 81 children were classified as maltreatment cases [159]. The authors showed using the novel PedBE clock that internalizing disorder in early childhood is associated with significantly accelerated E- ageing as opposed to children without internalizing disorder. The authors further demonstrated that the association between internalizing disorder and B-age is moderated by maltreatment severity and may be partly driven by glucocorticoids.

A study evaluated the relationship between prenatal maternal mental health and the PedBE clock [162]. Data were collected from two independent longitudinal cohorts from the Netherlands (*n* = 165) and Singapore (*n* = 340) on prenatal maternal anxiety [162]. Longitudinal evaluations of genome-wide DNAm derived from buccal cells were conducted at ages 6 and 10 in the Netherlands cohort and at 3, 9, and 48 months in the Singapore cohort. The findings revealed that prenatal maternal anxiety was linked to accelerated B-age in children using the Pediatric-Buccal-Epigenetic (PedBE) tool in both cohorts. This association was found to be more pronounced in males compared to females. Importantly, these results were independent of obstetric, socioeconomic, and genetic risk factors.

A study in Tianjin, China, sought to investigate whether exposure to prenatal gestational diabetes mellitus (GDM) could lead to accelerated B-age in the offspring at 3–10 years of age [163]. The study enlisted 578 mother–child pairs with gestational diabetes mellitus (GDM) and 578 non-GDM pairs. Horvath’s and Hannum’s clocks were employed to calculate DNA methylation (DNAm) age. Offspring born to women with GDM exhibited accelerated biological age (B-age) compared to control participants, regardless of other maternal factors. Moreover, the B-age of offspring was correlated with heightened cardiometabolic risk factors, indicating that GDM and its associated factors may have lasting effects on the biological age of offspring, with potential health implications.

The Extremely Low Gestational Age Newborn (ELGAN) study was a multi-center cohort study which enrolled ~1500 newborns born preterm (<28 weeks gestation) from 2002 to 2004 in five USA states (Connecticut, Illinois, Massachusetts, Michigan, and North Carolina) to examine epigenetic gestational age (eG-age) in the placenta [164]. The investigation explored the associations between placental epigenetic gestational age (eG-age) and various factors, including sociodemographic elements (maternal educational attainment, health insurance status, socioeconomic position, and maternal race), smoking, and clinical outcomes like Apgar scores and neonatal intensive care unit (NICU) length of stay. The Robust Placental Clock was employed to estimate eG-age for placental samples (*n* = 408) from extremely preterm infants. Maternal smoking, a known risk factor for preterm birth, was linked to positive placental eG-age acceleration, particularly among Black American mothers. In infants born to Black mothers, greater eG-age acceleration was observed compared to those born to white mothers. Additionally, placental eG-age acceleration was associated with shorter NICU lengths of stay, but this correlation was specifically noted among infants born to Black American women. This suggests that placental eG-age acceleration is influenced by sociodemographic factors and smoking, and exhibits variations among racialized groups.

Pregnancy can elicit stress-related disorders such as PTSD which could be associated with an increased risk of eG-age acceleration in newborns [165] and age acceleration in their mothers. A study was conducted to investigate the relationship between maternal stress exposure and PTSD symptoms with the mother’s own B-age and eG-age acceleration in the newborns (*n* = 89 maternal-neonatal dyads) [165]. Mothers were evaluated during the third trimester of pregnancy, and three epigenetic clocks—Horvath, PhenoAge, and GrimAge—were employed to examine the link between cumulative stress exposure in the previous year, prenatal post-traumatic stress disorder (PTSD) symptoms, and accelerated aging in women. DNAm data were derived from saliva samples collected from both mothers and neonates within 24 h of infant birth. The gestational epigenetic clock (EC) developed by Haftorn and colleagues based on the EPIC array was utilized in the analysis [166] to predict the gestational B-age of infants. Women who encountered a higher number of stressful life events, exhibited PTSD symptoms, and faced challenges in emotion regulation during pregnancy displayed accelerated GrimAge and PhenoAge soon after childbirth, indicating an elevated risk of premature mortality. Furthermore, infants born to mothers with higher PTSD symptoms during pregnancy exhibited lower epigenetic gestational age acceleration, suggesting slower development and an increased risk of later developmental issues.

A study was conducted in the UK to elucidate whether mothers’ adverse childhood experiences (ACE) (e.g., abuse, neglect, and household dysfunction) is associated with accelerated E-aging during pregnancy and in their newborns [167]. A total of 785 participants were included for maternal prenatal blood analysis, and 753 newborns were considered for umbilical cord blood analysis. DNAm was assessed in the mothers’ blood during pregnancy and in cord blood at birth. Biological age (B-age) acceleration was determined using clocks such as Horvath, Hannum, Skin-blood, GrimAge, PhenoAge, and DunedinPACE for the mothers, as well as the Knight and Bohlin cord blood clocks for the newborns. The study revealed a positive association between the mothers’ total Adverse Childhood Experiences (ACE) score and accelerated PhenoAge and GrimAge. In newborn males, the mothers’ ACE score was linked to accelerated epigenetic aging using the Bohlin clock. Moreover, male offspring born to mothers exposed to neglect showed accelerated B-age based on the Knight clock, and those exposed to parental substance abuse exhibited acceleration using the Bohlin clock. These findings suggest a connection between mothers’ ACE exposure and DNAm age acceleration in male offspring, highlighting the potential of DNAm age as a marker of intergenerational biological impact reflecting maternal childhood adversity.

The Drakenstein Child Health Study, a South African birth cohort, investigated the correlation between maternal psychosocial risk factors—specifically trauma/stressor exposure, PTSD, depression, and alcohol/tobacco use—and gestational biological age (B-age) at birth (*n* = 271 mother–child pairs) [168]. The eG-age at birth was determined using Bohlin’s epigenetic clock. The research revealed a negative association between maternal PTSD and child eG-age at birth, even after accounting for various confounding factors. The eG-age deviation at birth may hold clinical implications and contribute to an enhanced neurobiological comprehension of transgenerational trauma and PTSD.

## 4. Discussion

Molecular aging research has rapidly advanced in recent years due to the advent of biomarkers such as B-clocks. To elucidate aging as a risk factor for diseases and to design interventions, a quantitative measure such as a clock for biological age was required [169]. Epigenetic clocks have proven to be accurate at predicting chronological age and biological age. B-age, also commonly referred to as DNAm age, can differ from chronological age by many factors discussed in this review.

Some general limitations to take into consideration for the clocks are that G1 DNAm clocks were specifically designed to predict C-age. Therefore, they might not be good indicators for true ‘biological’ aging. G2 and G3 DNAm clocks were designed using various biomarkers/aging phenotypes. They might not predict ‘C-age’ accurately, but they were able to predict B-age with more precision. Some clocks were designed using data from small populations with limited diversity. These clocks might not be ideal predictors for populations comprising of diverse genetic and sociodemographic backgrounds. There are a limited number of clocks that were designed to work with children, and the interpretation of B-age in children is still not well understood. The technical variability and cell type composition of DNA samples used for analysis can influence the performance of some clocks. These variables should be accounted for in statistical analysis to maximize reliability. Furthermore, to increase reliability, it is recommended to use clocks with the tissue type/age group they were specifically designed for. Some clocks were designed with 450 K array data and do not perform as well with EPIC array data or other methods for measuring DNAm.

E-aging estimators have been increasingly used for non-medical applications by third parties, such as insurance companies, forensic scientists, and immigration officers to predict individual life expectancy [170]. However, this gave rise to several potential ethical, legal, and social conflicts. Multiple vulnerable groups of the population can be disproportionately affected by such usage [170]. For instance, decisions made by life insurance companies to mandate genetic testing may disproportionately impact families with a history of genetic disorders, diseases, BMI, cholesterol levels, and blood pressure. B-clocks have also been employed in determining the ages of refugees to differentiate minors from adults seeking asylum, particularly in cases where there are uncertainties about the validity of their identification documents. However, B-age estimators may not precisely distinguish between a 17-year-old minor and an 18-year-old adult. Previous medical conditions, which could either accelerate or decelerate E-aging, might influence the test results. Moreover, the collection of individual genetic and epigenetic information for immigration control purposes should adhere to widely recognized human rights principles, including the protection of privacy and human dignity [171]. Data from the B-clocks establish the links at the molecular level between social inequalities and health disparities. Therefore, scientists, bioethicists, social scientists, and human rights organizations should address the concerns regarding the use of epigenetics data and how it could be incorporated into international human rights law to help improve the health and life opportunities of socially disadvantaged individuals [170].

The discoveries of different types of B-clocks have therefore opened the avenue to study biological aging quantitatively. In this review, we have discussed the various B-clocks available, with their individual strengths and weaknesses, and how they are affected by different environmental exposures. Finally, the type of clock to be selected depends on the scientific query to be answered.

## 5. Future Perspectives

Several studies have pointed out the association between chronic stress and biological aging [153,172]. A promising area for future anti-aging interventions is to investigate whether accelerated biological aging caused by stress is reversible.

A crucial area for future exploration involves understanding how temporary increases in biological age and/or successful recovery from such changes may contribute to accelerated aging throughout life. From a technical perspective, it was observed across various human datasets that, when applied to the same dataset, first-generation (G1) DNAm clocks were incapable of detecting significant effects identified by second-generation (G2) clocks [173]. This could be due to the integration of multiple age-related biomarkers into the models of G2 clocks which makes them more sensitive to transient fluctuations in B-age compared with first-generation clocks, which are trained only on C-age.

Another interesting avenue to explore would be the development of B-clocks based on histone modifications. Post-translational modifications of histones can affect the aging process by modulating gene expression [174]. Histone methylation and acetylation at lysine residues are the most widely studied modifications known to affect the aging process [174]. Several studies indicate that global changes in H3K9me3, H4K20me3, H3K27me3, and H3K9ac levels occur during the process of aging [175]. However, histone modifications are less permanent than DNA modifications and have not been used in most biological clock models. RNA levels are an output of histone modifications and are easier to measure, which led to the development of RNA clocks [36]. For most B-clocks, the primary goal is to develop a reliable and ease-to-use and -screen biomarker. It is much easier to preserve samples (from human subjects or experimental animals or the field) for DNA isolation as opposed to sample preservation for later histone analysis. Moreover, methods for analyzing histone marks like mass spectrometry are also expensive [176].

In the past few years, a range of geroprotective drugs aimed at mitigating aging have undergone testing in animal models. These include compounds related to epigenetics (e.g., NAD+ precursors, sirtuin-activating compounds, and histone deacetylase inhibitors), small molecules with potent anti-diabetic effects (e.g., metformin), mTOR inhibitors (rapamycin), and antioxidant chemicals (N-acetyl-l-cysteine) [174]. Metformin exerts its effects through extensive epigenetic regulation. Numerous studies suggest that it enhances both the life and health span of mice, demonstrating efficacy even when administered in middle age (12 months) or old age (20–24 months) [177,178]. Rapamycin, on the other hand, prolongs the lifespan in mice through multiple mechanisms. These include reducing DNAm marks in the hippocampus to slow brain aging, alleviating aging-related epigenetic signatures in mouse livers, and mitigating various aging-related diseases such as cardiovascular dysfunction, neurodegeneration, hearing loss, skeletal muscle aging, ovarian aging, and more [179,180,181,182]. Some of these anti-aging drugs are now extensively tested in clinical trials [174].

In summary, aging research has influenced almost all aspects of biological and clinical sciences. Future efforts to understand B-aging and age reversal in the field of toxicogenomics will have an enormous effect on human lifespan and health span.

## Figures and Tables

**Table 1:** An overview of different epigenetic clocks.

Type of Clock		Name of the Clock	Publication Year and Ref.	# of C_p_G	No. of Subjects (N)	Age Range	Tissue	Training Phenotype	Platform
DNAm -based molecular epigenetic clocks	G1 clocks	Horvath’s clock	2013; [22]	353	7844	0–100	51 healthy tissues & cell types	C-age	Illumina 27 K and 450 K
		Hannum’s clock	2013; [23]	71	482	19–101	Whole blood	C-age	450 K
	G2 clocks	DNAm PhenoAge	2018; [30]	513	9926	21–100	Multiple	Lifespan (mortality risk score)	27 K and 450 K and EPIC
		DNAm GrimAge	2019; [31]	1030	6935	46–78	Whole blood	Lifespan (mortality risk score)	450 K and EPIC
	G3 Clocks	Dunedin PACE	2020; [32]	46	810	26–38	Whole Blood	Pace of Aging	450 K and EPIC
DNAm- based mitotic clocks		EpiTOC	2016; [33]	385	656	19–101	Whole blood	Mitotic age, cancer risk	450 K
		MiAge	2018; [33]	286	4020	N/A	8 TTGA Cancer cells	Mitotic age, cancer risk, survival	450 K
		EpiTOC2	2020; [34]	385	656	19–101	Whole blood	Mitotic age, cancer risk	450 K
Sperm epigenetic clocks		Sperm epigenetic aging (SEA)CpG clock	2022; [35]	803,063	379 semen samples	≥18	Semen	Sperm epigenetic aging	EPIC Infinium Methylation Beadchip
RNA clocks		RNAAgeCalc	2020; [36]	353	9448 samples	N/A	Multi-tissue	Transcriptional age	RNA-Seq data from the Genotype-Tissue Expression (GTEx) Program
		MultiTIMER	2023; [37]	N/A	23,000 annotated samples	N/A	Multi-tissue	C-age	RNA-seq samples (ArchS4) v11
Single-cell epigenetic clock framework (scAge)		scAge	2021; [38]	500,000 CpGs/ cell	549 tissue samples	1–21 months old mice	Multi-tissue	Single-cell B-age predictions	Computational platform
Pediatric epigenetic clocks		Knight’s clock	2016; [39]	148	207	24–42 weeks	Cord blood	Gestational Age	27 K and 450 K
		Bohlin’s clock	2016; [40]	132	685	Neonates	Cord blood	Gestational Age	450 K
		Lee’s placental clock	2019; [41]	441,870	1102	5–42 weeks	Placenta	Gestational Age	450 K and EPIC
		Pediatric-Buccal-Epigenetic (PedBE) clock	2020; [42]	94	1032	0 to 20 years old	Buccal epithelial cels	Pediatric age	450 K and EPIC
		Mayne’s clock	2017; [43]	62	409	8–42 weeks	Placenta	Gestational age	27 K and 450 K
		NeoAge clocks (4 epigenetic clocks)	2021; [44]	303–522	542	Pre-term infants (<30 weeks)	Buccal cell samples	Post-menstrual and postnatal age of neonates	450 K and EPIC

## Data Availability

Not Applicable.
